# Characterization of substance use disorder during pregnancy in a syphilis population

**DOI:** 10.1002/pmf2.70094

**Published:** 2025-08-28

**Authors:** Kristen Warncke, Alesha White, Jessica E. Pruszynski, Scott Roberts, Vanessa Rogers, Emily H. Adhikari

**Affiliations:** ^1^ Department of Obstetrics & Gynecology University of Texas Southwestern Medical Center Dallas Texas USA; ^2^ Parkland Health Hospital System Dallas Texas USA

**Keywords:** methamphetamine, opioids, substance use disorder, syphilis

## Abstract

**Introduction:**

Rates of congenital syphilis and substance use disorder (SUD) have concurrently risen in pregnant patients. Specific substances and their association with inadequately treated syphilis have not yet been identified. The objective of our study was to characterize SUD, including specific drugs of use, among pregnant people with syphilis to determine the association between SUD and adequacy of syphilis treatment.

**Methods:**

Patients diagnosed with syphilis during pregnancy between January 1, 2021 and December 31, 2022 were identified. SUD was identified via chart review and confirmed or supported by the use of universal screening for self‐reported SUD, ICD‐10 codes for mothers and neonates, and toxicology. Maternal demographics and delivery and neonatal outcomes were compared among those with and without SUD. Logistic regression models adjusted for maternal age, Black race, nulliparity, and hypertension were used to produce odds ratios relating SUD to delivery and neonatal outcomes. The timing of penicillin treatment initiation less than 30 days versus 30 or more days before delivery in individuals with and without SUD was compared.

**Results:**

Of 225 patients with syphilis during pregnancy, 40 (17.8%) had SUD. Compared to those without SUD, those diagnosed with SUD were more likely to be Non‐Hispanic White with late and limited prenatal care. They were almost six times more likely to have preterm delivery (aOR 5.98 [95% CI 2.47–14.79]) and three times more likely to develop pre‐eclampsia with severe features (aOR 3.48 [95% CI 1.41–8.41]). The most common primary drugs of use included non‐prescription opioids (32%), methamphetamines (32%), and cannabis (20%). Frequency of syphilis treatment initiation less than 30 days before delivery was higher in individuals with SUD (52% vs. 21%, *p* = 0.001). Methamphetamine was the most common primary drug of use among patients with SUD who were inadequately treated for syphilis and its rate of use was significantly higher when compared to patients with SUD who were adequately treated (9/21 [43%] vs. 4/19 [21%], *p* = 0.041).

**Conclusions:**

Pregnant people with SUD, and specifically use of methamphetamines, were more likely to have inadequate syphilis treatment than those without SUD. In addition, demographic and prenatal characteristics differed between individuals with and without SUD, with poorer delivery outcomes observed in patients with SUD and syphilis compared to a syphilis population alone. Future research is needed that addresses the impact of medication‐assisted therapies for the treatment of methamphetamine use on rates of adequate syphilis treatment.

## 1 INTRODUCTION

Although congenital syphilis is preventable with adequate maternal treatment, rates of congenital syphilis have increased 701% from 2012 to 2021 in the United States [1]. According to the Centers for Disease Control and Prevention (CDC), over 10 times as many babies were born with syphilis in 2022 when compared to 2012 [2]. Concurrently, rates of substance use disorder (SUD) have increased in pregnant patients. According to the 2022 National Survey on Drug Use and Health (NSDUH), between 2021 and 2022, rates of substance use among pregnant female adults between the ages of 15 to 44 rose from 7.9% to 9.6% [3]. In addition, according to the CDC, the number of women with opioid related diagnoses at delivery hospitalization increased by 131% from 2010 to 2017 [4, 5]. In certain states, overdose is the leading cause of pregnancy‐related death in the year following delivery and according to the 2017–2019 Maternal Mortality Review Committee findings, mental health conditions to include deaths due to overdose or poisoning‐related to SUD made up the largest percentage of maternal deaths at 23% in a summary of 36 states [6, 7, 8].

Stigma associated with SUD in pregnancy in combination with fear of being reported results in a lack of prenatal care in this population [9]. Limited prenatal care in pregnant patients with syphilis results in delay of adequate treatment of maternal syphilis defined as treatment initiated less than 30 days before delivery [8, 9, 10, 11, 12, 13, 14]. This relationship of syphilis with substance use and inadequate prenatal care is recently recognized [7], but underexplored in the context of specific substances and how they may be more or less strongly associated with untreated syphilis in pregnancy. The primary objective of this study was to characterize SUD, including specific drugs of use among pregnant patients with syphilis, and to determine the association between SUD and adequacy of syphilis treatment. Secondary outcomes were to describe maternal and neonatal outcomes in a population of pregnant people with syphilis with and without SUD.

## 2 METHODS

We conducted a retrospective cohort study of individuals with reactive reverse sequence treponemal screening from January 1, 2021 through December 31, 2022 who delivered at our institution. Reverse sequence screening is performed at our institution at the first prenatal visit, in the third trimester, and at delivery, which is consistent with the testing schedule required by the state in which this study was conducted. Initial screening is performed with a treponemal chemiluminescence immunoassay (CIA), followed by a non‐treponemal antibody, the rapid plasma reagin (RPR). An RPR titer is then performed, which is used with clinical evaluation to make the diagnosis of active or past syphilis, and for monitoring of serologic response to treatment. Among those with discordant testing (CIA+, RPR−), a second treponemal test is performed for confirmation, the *Treponema pallidum* particle agglutination (TP‐PA) test [15, 16, 17]. If reactive, a diagnosis of active or past syphilis is made. Clinical history and physical exam determine whether individuals with abnormal syphilis screening have a false positive antibody, new or active infection that warrants treatment, past infection without need for treatment, or suspected reinfection. At our institution, standardized protocols are followed to refer ambulatory patients with abnormal syphilis screening to a centralized maternal fetal medicine (MFM) clinic, where providers complete clinical history and staging evaluation under the direct supervision of MFM faculty with expertise in infectious diseases in pregnancy.

SUD within our syphilis population was identified based on documentation of SUD in the electronic medical record (EMR) by a healthcare provider with formal assessment by a specialized multidisciplinary team known as the Perinatal Intervention Program (PIP) when available. This specialized team provides comprehensive treatment and recovery services during pregnancy in both the inpatient and outpatient settings. Starting in June 2021, all patients within our prenatal care system started undergoing universal screening with the National Institute of Drug Abuse (NIDA) Quick screen V1.0 at the first prenatal visit. Screening results, ICD‐10 codes, and toxicology screens for mothers and neonates were used secondarily to support the diagnosis of SUD, as previously described [18].

Medical record review was conducted to determine maternal demographics, characterize syphilis staging, initial non‐treponemal titer, and pregnancy and neonatal outcomes among those with and without SUD in pregnancy. The timing of penicillin treatment initiation less than 30 days (considered inadequate) versus 30 or more days before delivery was compared among individuals with and without SUD. Adequate timing of treatment for syphilis was considered 30 or more days before delivery based on CDC recommendations [2]. We characterized pregnant patients with syphilis with and without SUD and compared the primary drug of use in the overall cohort and in subgroups based on syphilis treatment timing. This study was approved with a waiver of consent by the Institutional Review Board at our institution.

### 2.1 Statistical analysis

Chi‐square test and Fisher's exact test were used to compare categorical variables between the groups. Student's *t*‐test was used to compare normally distributed continuous variables and the Kruskal‐Wallis test was used to compare non‐normally distributed continuous variables not normally distributed. Logistic regression models were used to produce odds ratios relating SUD to delivery and neonatal outcomes. The models were adjusted for maternal age, Black race, nulliparity, and hypertension. Statistical analyses were performed in R version 4.40 [19]. A *p* value less than 0.05 was considered statistically significant.

## 3 RESULTS

From January 1, 2021 through December 31, 2022, 225 individuals were diagnosed with syphilis during pregnancy by reverse sequence screening and clinical staging. Of these patients, 40 (17.8%) had documented SUD. Compared to those without SUD, those with SUD were more likely to be older (mean age 29 ± 5.7 vs. 26.7 ± 6, *p* = 0.024), Non‐Hispanic White (10 [25%] vs. 14 [8%], *p* = 0.004), and have late and limited prenatal care. They had fewer median prenatal care visits (2 [0–8] vs. 10 [6–14] visits, *p* < 0.001) and initially presented at later median gestational ages (24 [13–33] vs. 16 [10–25] weeks, *p* = 0.022). Although they presented at later gestational ages for their first RPR titer (median gestational age 30 weeks [23–35 weeks] vs. 17 weeks [10–28 weeks], *p* < 0.001), there was no difference in initial RPR titers or syphilis stage, with the majority having latent syphilis (late or unknown duration) (37 [92%] vs. 158 [85%], *p* = 0.231) (Table 1).

**TABLE 1:** Maternal demographic and prenatal characteristics in a pregnant patient with syphilis with and without concomitant SUD, January 1, 2021 through December 31, 2022.

Outcome	SUD (*n* = 40)	No SUD (*n* = 185)	*p* value
Age	29 (5.7)	26.7 (6.0)	0.024
Race/ethnicity			0.004
Black, Non‐Hispanic	10 (25)	88 (48)	
White, Non‐Hispanic	10 (25)	14 (8)	
Hispanic	20 (50)	80 (43)	
Other	0 (0)	3 (2)	
Nulliparous	8 (20)	62 (34)	0.09
Prenatal care visits	2 (0–8)	10 (6–14)	< 0.001
GA at first prenatal visit^a^	24 (13–33)	16 (10–25)	0.022
RPR #1 at diagnosis, titer			0.64
< 1:8	8 (20)	50 (27)	
> 1:8	23 (57)	99 (54)	
Nonreactive	9 (22)	36 (19)	
GA at RPR #1	30 (23–35)	17 (10–28)	<0.001
Stage of syphilis			0.23
Early (primary, secondary, NPNS)	3 (8)	27 (15)	
Latent, unknown duration or late	37 (92)	158 (85)	
HIV Ag/Ab reactive	2 (5)	8 (4)	0.69
Early pregnancy loss	0 (0)	0 (0)	
In CHNA high‐deprivation zip code	12 (30)	48 (26)	0.59
Distance to first prenatal clinic, miles^a^	12.3 (9.0–14.8)	9.3 (4.3–14.8)	0.19
Timing of syphilis treatment			
< 30 days prior to delivery	21 (52)	38 (21)	0.001
≥30 days prior to delivery	19 (48)	147 (79)	0.018

*Note*: Data shown as *n*(%), mean ± SD or median (Q1–Q3) as appropriate.

Abbreviations: CHNA, Community Health Needs Assessment; GA, gestational age; NPNS, non‐primary non‐secondary; SUD, substance use disorder.

^a^ Among individuals with prenatal care.

Patients with SUD were almost six times more likely to have preterm birth defined as birth at less than 37 weeks (OR 6.18 (95% CI 2.73–14.06)) and four times more likely to have preterm birth defined as less than 34 weeks (OR 4.39 (95% CI 1.20–15.38). This remained true after adjusting for maternal age, Black race, nulliparity, and hypertension (aOR 5.97 [95% CI 2.47–14.79] and aOR 5.33 [95% CI 1.36–20.87]). Patients with SUD were also three times more likely to develop pre‐eclampsia with severe features (OR 3.31 [95% CI 1.40–7.62]). This also remained true after adjusting for maternal age, Black race, and nulliparity (aOR 3.48 [95% CI 1.41–8.41]). The most common diagnostic criteria met for diagnosis of pre‐eclampsia with severe features in both cohorts were severely elevated blood pressures (55% in the SUD cohort and 68% in the cohort without SUD) followed by proteinuria (27% in the SUD cohort and 32% in the cohort without SUD). As some patients had multiple criteria met for diagnosis of pre‐eclampsia with severe features, these percentages could not be compared statistically but did not clinically appear to be different. The rate of stillbirth was higher in patients with SUD (2 [5%] vs. 0 [0%]) but an odds ratio could not be calculated. There were no differences in rates of cesarean delivery, chronic hypertension, gestational or overt diabetes, or chorioamnionitis (Table 2).

**TABLE 2:** Pregnancy outcomes of patients with syphilis, with and without substance use disorder.

Outcome	SUD (*n* = 40)	No SUD (*n* = 185)	*p* value	Unadjusted OR	Adjusted OR
GA at delivery	38 (35–39)	39 (38–40)	< 0.01	—	—
GA < 37 weeks	15 (38)	17 (9)	< 0.001	6.18 (2.73–14.06)	5.98 (2.47–14.79)^a^
GA < 34 weeks	5 (12)	6 (3)	0.026	4.39 (1.20–15.38)	5.33 (1.36–20.87)^a^
Cesarean delivery	21 (52)	76 (41)	0.19	1.59 (0.80–3.17)	1.81 (0.85–3.88)^a^
Cesarean for fetal indication	3 (8)	22 (12)	0.58	0.60 (0.14–1.85)	0.89 (0.19–3.09)^a^
Chronic hypertension	2 (5)	12 (6)	> 0.99	0.76 (0.12–2.93)	0.83 (0.12–3.52)^b^
Pre‐eclampsia	11(28)	19 (10)	0.004	3.31 (1.40–7.62)	3.48 (1.41–8.41)^b^
Gestational diabetes^c^	1 (2)	13 (7)	0.47	0.34 (0.02–1.78)	—
Overt diabetes^c^	1 (2)	3 (2)	0.55	1.56 (0.08–12.51)	—
Chorioamnionitis	4 (10)	21 (11)	> 0.99	0.87 (0.24–2.45)	1.01 (0.27–3.06)^a^
Stillbirth^c^	2 (5)	0 (0)	0.031	—	—

*Note*: Data shown as *n*(%), mean ± SD, or median (Q1–Q3) as appropriate.

Abbreviations: GA, gestational age; OR, odds ratio; SUD, substance use disorder.

^a^ Odds ratio adjusted for Black race, maternal age, nulliparity, and hypertension.

^b^ Odds ratio adjusted for Black race, maternal age, and nulliparity.

^c^ Event rate too low to calculate odds ratio.

Neonates born to mothers with syphilis and SUD during pregnancy had longer median hospital stays (14 [11, –23] vs. 4 [3–11] days, *p* < 0.001) and were more likely to require intravenous (IV) penicillin treatment for 10 (25 [62%] vs. 61 [32%], *p* < 0.001) and 14 days (3 [8%] vs. 0 (0%), *p* < 0.001). Neonates born to patients with SUD were three times more likely to receive IV penicillin treatment for 10 days (OR 3.52 [95% CI 1.75–7.30]) and this remained true after adjusting for maternal age, Black race, and nulliparity (aOR 3.21 [95% CI 1.57–6.75]). Odds ratios for treatment with IV penicillin for 14 days could not be calculated due to the low event rate in both groups (Table 3).

**TABLE 3:** Neonatal outcomes among liveborn infants exposed to syphilis, with and without substance use disorder.

Outcome	SUD (*n* = 40)	No SUD (*n* = 190)	*p* value	Unadjusted OR	Adjusted OR^a^
Neonatal length of stay^b^	14 (11–23)	4 (3–11)	< 0.001		
Neonatal treatment			< 0.001		
Benzathine penicillin IM	12 (30)	126 (66)		0.22 (0.10–0.45)	0.22 (0.10–0.47)
IV Penicillin ×10 days	25 (62)	61 (32)		3.52 (1.75–7.30)	3.21 (1.57–6.75)
IV Penicillin ×14 days^c^	3 (8)	0 (0)		—	—
Not treated^c^	0 (0)	1 (1)		—	—
Neonatal demise^c^	0 (0)	2 (1)		—	—

*Note*: Data shown as *n*(%), mean ± SD, or median (Q1–Q3) as appropriate.

Abbreviations: GA, gestational age; IM, intramuscular; IV, intravenous; OR, odds ratio; SUD, substance use disorder.

^a^ Odds ratio adjusted for Black race, maternal age, and nulliparity.

^b^ Reported in days.

^c^ Event rate too low to calculate odds ratio.

### 3.1 Substance use characteristics

**FIGURE 1:**
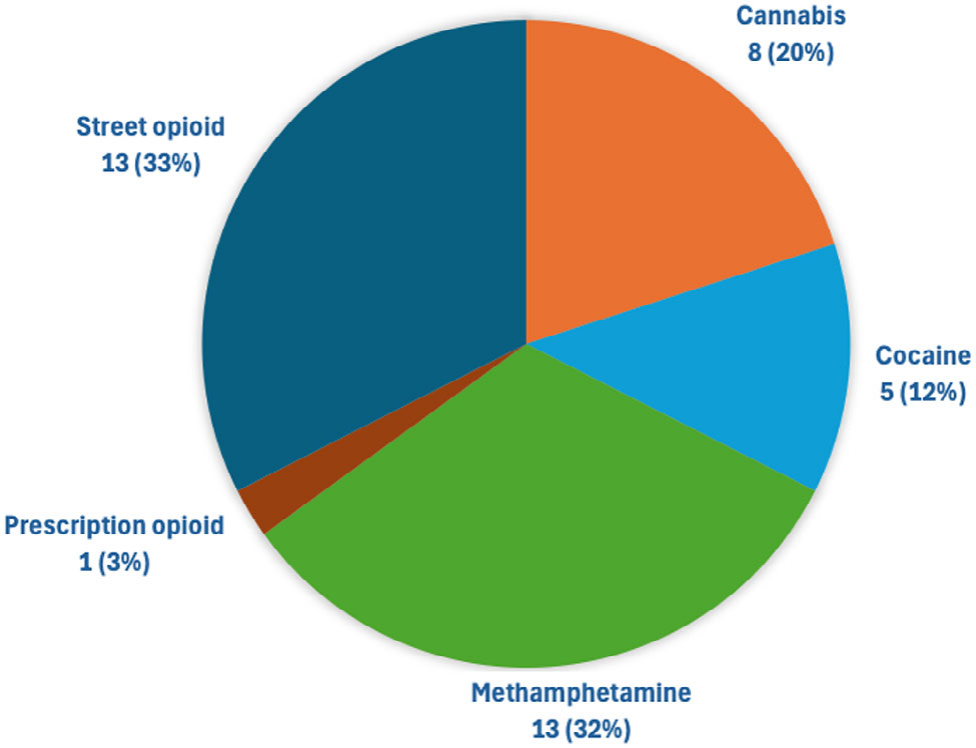
Primary drugs of use among 40 patients with syphilis and concomitant substance use disorder.

**FIGURE 2:**
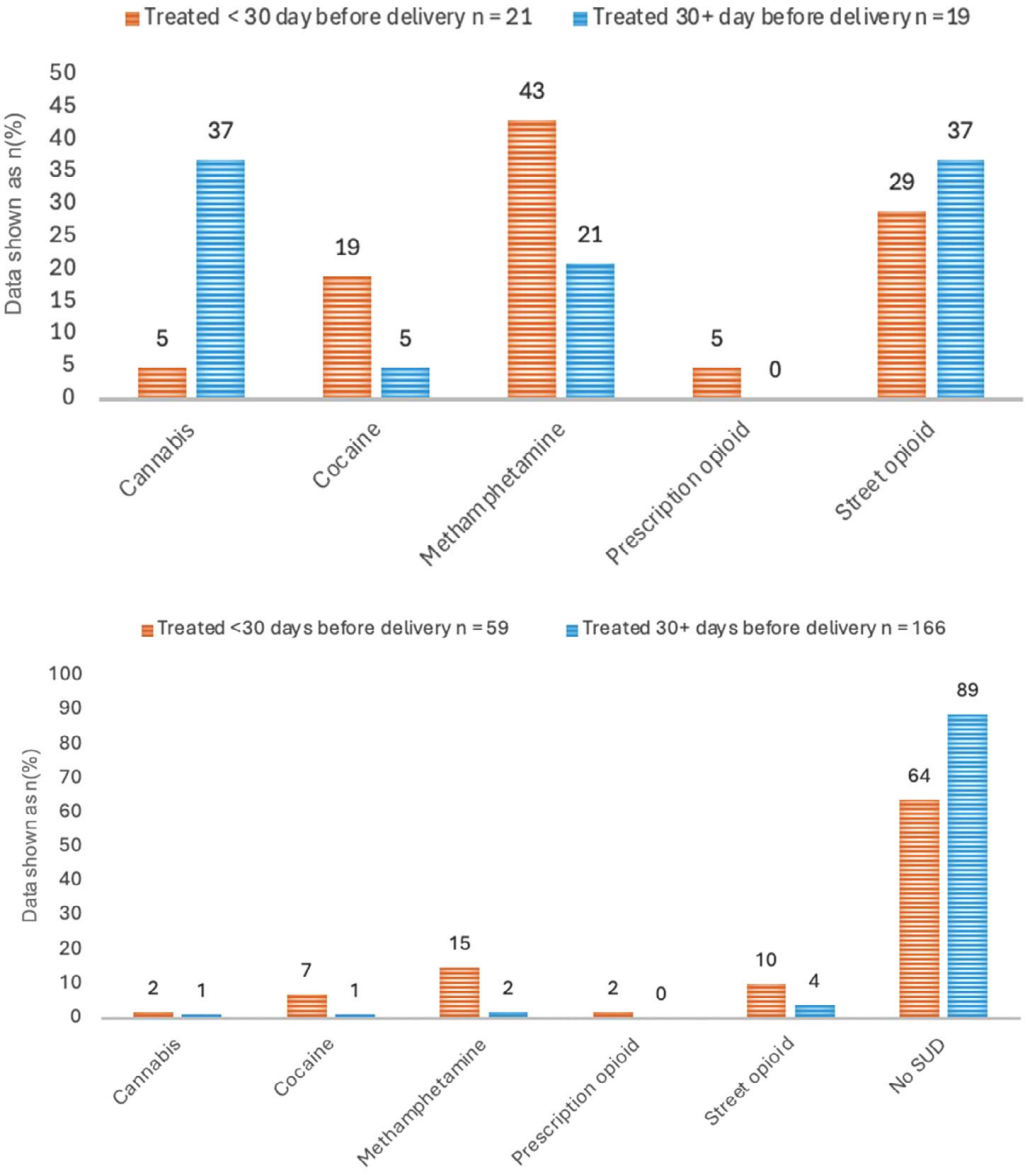
Primary drugs of use among pregnant patients with syphilis and SUD (*N* = 40) based on timing of treatment < 30 or 30 or more days before delivery.

Compared to those without SUD, patients with SUD were more likely to have inadequate syphilis treatment (21 [52%] vs. 38 [21%], *p* = 0.001) (Table 1). The most common primary drugs of use for patients with SUD and syphilis were non‐prescription opioids (13 [32.0%]), methamphetamines (13 [32.0%]), and cannabis (8 [20.0%]) (Figure 1). Primary drugs of use varied based on the timing of treatment. Patients with SUD with inadequate syphilis treatment were more likely to use methamphetamines than patients with SUD with adequate syphilis treatment (9/21 (43%) vs. 4/19 (21%), *p* = 0.041) (Figure 2).

## 4 DISCUSSION

Of pregnant patients with syphilis at our institution, 18% have SUD. Despite uniform referral and treatment services for syphilis in pregnancy, rates of inadequate treatment are nearly two times higher in patients with SUD. Patients with SUD were seen fewer times in the prenatal care system and had higher rates of preterm birth. Their neonates had significantly longer admissions and received longer durations of IV penicillin treatment. Finally, methamphetamines were the most common primary drug of use for patients with inadequate syphilis treatment, with almost half of inadequately treated patients with SUD reporting methamphetamine use.

Our findings in this study are consistent with previous studies demonstrating high rates of limited or no prenatal care among pregnant individuals with SUD and thus increased maternal and neonatal morbidity [7, 9, 11, 18]. Additionally, previous data have shown that SUD alone poses substantial risks, including fatal overdose, fetal growth restriction, preterm birth, and neonatal abstinence syndrome [4, 5]. Higher rates of preterm birth specifically are seen in our study in those with both syphilis and SUD. Whether syphilis infection exacerbates or increases risk for preterm birth in patients with SUD is unexplored. Although these previous findings were confirmatory of what is already known, a new finding, not previously reported, was found between the relationship of pre‐eclampsia with severe features and SUD. While higher rates of pre‐eclampsia with severe features in individuals with SUD may be secondary to the physiologic effects of illegal drugs on blood pressure, further study is needed to elucidate mechanisms of preeclampsia and preterm birth and the potential dynamic interaction between infection, SUD, and pre‐eclampsia at the placental level.

Many studies have been completed that look at barriers to care for patients with syphilis and have highlighted the relationships of limited prenatal care and SUD separately to overall reduced syphilis treatment rates [7, 11]. Data on newly diagnosed syphilis in pregnancy have shown that limited prenatal care, further distance traveled to prenatal care clinics, and being concentrated in zip codes with high deprivation contribute to delays in treatment [20, 21, 22]. Studies that specifically evaluate characteristics of patients with syphilis and SUD are sparse. Carlson et al. is one of the few studies that have reported on patients with SUD and syphilis. This study reported on the prevalence of SUD and its relationship to rates of congenital syphilis in patients in Arizona and Georgia between 2018 and 2021 [7]. This study found that rates of SUD were twice as high in the congenital syphilis cohort and highlighted higher rates of lack of prenatal care in the SUD population. One‐third of patients in this analysis with SUD remained untreated for syphilis. Our study reports similar findings with one‐third of our syphilis population with SUD receiving delayed treatment for syphilis. Although similar in this regard, our study further adds to this data by providing a detailed analysis of patient characteristics and the association between the type of substance used and the timing of treatment for syphilis in pregnancy. However, further research is needed to identify barriers to care for patients with SUD and syphilis in pregnancy.

Established medication assistance programs with the use of US Food and Drug Administration–approved opioid agonist pharmacotherapy have been successful in the treatment of opioid use disorder in pregnancy [17]. However, less is understood about the treatment of other substance use disorders in pregnancy, including treatment for methamphetamine, cannabis, or cocaine use. This study highlights that further research on medication assistance treatment for methamphetamine use disorder in pregnancy and its effect on the rate of adequate syphilis treatment is much needed.

Strengths of our study include that this study was completed at a large single institution from a large public health region in a state in which almost half of all congenital syphilis cases in the United States occurred in 2020 [6]. There was both a robust sample population of patients with syphilis and SUD. Additionally, access to treatment with penicillin G benzathine (IM) for syphilis is prioritized for pregnant individuals at our institution despite shortages and is provided free of charge for all patients [21, 24, 25]. Another strength of this study includes the systematic testing algorithm, clinical identification, and referral to a centralized MFM clinic for both SUD and syphilis. During this study, we also implemented a universal screening questionnaire in the setting of an already well‐established referral process to our specialized prenatal clinic to increase rates of SUD detection and treatment.

Limitations include potential misdiagnosis or missing information on SUD in the medical record and the retrospective nature of this study. Universal screening for substance use was not started at our institution until June 2021. There is a possibility that without the implementation of universal screening, we could have missed patients with SUD who would have screened positive with the NIDA quick screen from January to June 2021. Additionally, criteria for diagnosis via the Diagnostic and Statistical Manual of Mental Health – 5 (DSM ‐ 5) was not routinely documented in the charts of patients with SUD, thus making it difficult to use this as a diagnostic criterion retrospectively. This could have led to possible overdiagnosis of SUD based on retrospective chart review alone. Further studies are needed that analyze placental pathology and neonatal clinical findings to elucidate the role of SUD in congenital syphilis associated outcomes in this specific population. Additionally, this is an observational study and thus the findings in this study are associations that cannot be directly tied to causation. Lastly, our prenatal care population may not be generalizable to other prenatal populations with differing rates of SUD and/or syphilis.

## 5 CONCLUSION

Despite uniform referral and treatment services, rates of inadequate treatment of syphilis among pregnant people with concomitant syphilis infection and SUD remain high. In addition, demographic and prenatal characteristics differ between individuals with and without SUD in a population with syphilis. Early identification of SUD and syphilis as well as efforts to increase access to prenatal care are important to remove barriers to adequate maternal syphilis treatment. Specifically, future research focused on medication‐assisted therapies that target methamphetamine misuse and their influence on rate of adequate syphilis treatment are urgently needed.

## CONFLICT OF INTEREST STATEMENT

The authors report no conflicts of interest.
